# A 3D computer-assisted treatment planning system for breast cancer brachytherapy treatment

**DOI:** 10.1007/s11548-014-1092-y

**Published:** 2014-07-08

**Authors:** Yuchong Rachel Jiang, Edward R. Sykes

**Affiliations:** Faculty of Applied Science and Technology, Sheridan College Institute of Technology and Advanced Learning, 1430 Trafalgar Road, Oakville, Ontario Canada

**Keywords:** Visualization, Breast brachytherapy, 3D treatment planning system

## Abstract

**Purpose:**

Brachytherapy is an option for treatment of breast cancer in some cases. This modality requires patient-specific dosimetry based on CT simulator scans. A 3D computer-assisted breast brachytherapy treatment planning system called Vision was developed and tested.

**Methods:**

The brachytherapy treatment planning system used volume estimation and dose analysis with advanced 3D visualization. The patient treatment volume reconstruction was designed to ensure high-volume accuracy requirement of radioactive seed implantation procedure for this treatment. The system enables interactive placement of radioactive seeds embedded in original patient CT images with 3D display.

**Results:**

The system achieved 99.73 % accuracy in volume estimation measured against the true volume and is statistically significantly more accurate than current existing commercial software at the $p=0.05$ level.

**Conclusion:**

A virtual 3D environment was developed to perform volume measurements, seed placements, and dose distribution planning and analysis based on 2D contours on patient CT images. This system was demonstrated to be feasible and accurate in a clinical setting.

## Introduction

Breast cancer is the most common type of cancer in Canadian women [1, 2]. Every year in Ontario, about 7,800 women undergo surgeries to remove cancer from their breasts [3–5]. Radiation therapy has been established as an effective adjuvant therapy for breast cancer. With radiation therapy, patients no longer need to undergo mastectomy to remove the whole breast [6]. Instead, only a small part of the breast needs to be removed, and the rest is treated by radiation therapy. Lumpectomy and whole breast radiation achieve the same survival rate as mastectomy, but offers much better cosmetic results and improved health-related quality of life [2, 7, 8]. Brachytherapy has emerged as a newer technique that may offer similar control rates as whole breast radiation with the additional benefits such as shorter treatment days and improved convenience [9, 10]. Another benefit of brachytherapy for breast cancer treatment is that it has the potential to overcome the problems associated with external beam radiation therapy, such as burning of the skin [9–11]. In brachytherapy, radioactive sources are implanted directly into the breast.

At the Toronto-Sunnybrook Regional Cancer Center, several software packages for treatment planning were analyzed, such as Pinnacle and Varian as well as some manual intervention procedures [2, 4, 12]. Furthermore, several software solutions for prostate cancer brachytherapy treatment were also investigated [12]. These systems use ultrasound guidance and have been shown to be very successful. However, these techniques are not suitable for breast brachytherapy because the nature of operation for breast is inherently different than for other organs; furthermore, there are no existing software solutions entirely suitable for this type of treatment [2, 7].

Consequently, we sought out to design and build a system to overcome these shortcomings for breast cancer brachytherapy. A unique system is specifically tailored for breast cancer brachytherapy treatment planning. This 3D virtual simulation environment can be used for seed placement planning, dose analysis, seeds location and spacing examination, modification, visualization, and position verification. This paper presents a report on this new system called *Vision* that was designed, developed, and refined for breast cancer brachytherapy treatment planning. *Vision* is based on the technique of adjuvant partial breast irradiation, uses $^{103}$Pd permanent seed implants, and was developed at the Toronto-Sunnybrook Regional Cancer Center [6]. The system achieves 99.73 % accuracy in volume estimation measured against the true volume and is statistically significantly more accurate than current existing commercial software at the $p=0.05$ level.

## Literature review

Brachytherapy is one of the more well-known methods for cancer treatment that involves placing radioactive sources directly into or next to the area requiring treatment [13]. This enables clinicians to deliver a high dose with minimal impact on surrounding healthy tissues. Brachytherapy has been shown to be a highly successful treatment for cancers of the prostate, cervix, endometrium, breast, skin, bronchus, esophagus, and head and neck, as well as soft tissue sarcomas and several other types of cancer [12–15].

Over the last decade, increasing numbers of breast cancer patients are being treated using interstitial radioactive implants [4, 12, 15]. Systems have been created to assist in the planning for brachytherapy treatments that provide a visualization environments based on CT/MRI images [1, 7, 11, 13, 16]. These systems provide doctors with an interactive source layout function, including accurate and quick dosage distribution calculation and dynamical 2D/3D display. These systems also provide features such as data acquisition, registration, segmentation, image processing, 3D reconstruction, and planning report output. Clinical practice has shown they meet the therapeutic demands. However, in these systems, there are deficiencies in the features of the treatment planning interface, validation, and verification modules and in the accuracy of the volume calculation—particularly for the breast treatment [4, 12, 13].

One of the more widespread systems used is GZP6—a high dose rate (HDR) brachytherapy system [2]. In their system, the interface deficiencies identified above were addressed. Their system incorporates an improved treatment planning visual interface component called CTPS [2]. The authors claim that it is an essential component for evaluating the correctness of non-predefined treatments and is easy use for medical staff [2]. Their system is limited; however, in that, their system provides only a partial solution for the planning breast treatments [2]. Other software systems exist but do not allow for virtual planning [1, 2, 11, 12].

In summary, general solutions for brachytherapy are known; however, there are deficiencies in each of these systems (e.g., tools, planning modules, verification modules, etc.) [1, 2, 4, 11–13, 15]. These existing solutions do not adequately provide the full range of tools or support for oncologists to effectively and accurately treat breast cancer patients. There is a need for a comprehensive solution for breast brachytherapy. Such a system is presented in this paper. In our system, called *Vision*, an entire 3D treatment planning and verification solution are provided.

## Methods

In the design and development of *Vision*, the following method was used:

- reading CT data set, Region of Interests (ROIs), and RT plans from Pinnacle;
- reslicing the CT data, ROIs, and RT to the oblique direction;
- reconstructing the volume with high resolution;
- computing volume interpolation for accurate target volume measurement;
- simulating the fiducial needle and providing surgical templates;
- facilitating radiation source editing by designing a customized editor for managing $^{103}$Pd source model data;
- determining a template size for the seed implantation—usually the template must cover the whole area of the ROIs;
- superimposing the simulated template onto each image that has ROIs along the gantry angle;
- providing an interface for placing needle/seeds based on the superimposed template;
- calculating the radial dose distributions on the seeds placed by computer simulation to avoid hot/cold spots to ensure a uniform dose distribution that will cover the entire Planned Target Volume (PTV) by adjusting source activity and the number/position of needles/seeds;
- projecting the seeds back to the original image data set for 3D viewing.

### Data collection and processing

The data collection phase involves a CT scanner on which the patient lies in prone position. The CT images are taken around the chest region traveling in the direction from the head to the feet. The $Z$ spacing is 5 mm away from the breast and 2 mm on the breast region. Oncologists manually determine the PTV, clinical target volume (CTV), and ISO center and a gantry angle on each slice around the cavity region where the tumor was removed. The gantry angle is parallel with the rib cage to protect needles from going through the rib and lung region during the operation. The oblique reslicing direction is perpendicular to the gantry angle. Seed placement is carried out on the resliced images inside the PTV contours that are reconstructed in the oblique direction. The number of interpolations between two adjacent slices is determined by the distance between them. For example, if the distance is 5 mm, then four interpolations are computed. Our interpolation method guarantees uniform unit spacing between slices.

### Reslicing images and ROIs

The reslicing task is done by projecting the data set on the direction perpendicular to the gantry angle after applying interpolation on the CT images and its ROIs. The new reconstructed data can be described as follows:

- the $X$ direction is parallel with the original $Z$ direction
- the $Y$ direction is same as the original $Y$ direction with an angle that is derived from the gantry angle.

From posterior to anterior:

- the $Z$ direction parallel with the original $X$ direction with an angle equal to the gantry angle.

The total number of resliced slices is automatically calculated using the ROIs size along the gantry angle and is described by the following equation:

$$\begin{aligned} \mathrm{Total\,number}_{\mathrm{reslices}} =\left( \frac{\mathrm{ROI\,size\,along\,gantry\,angle}}{\mathrm{\,reslice\,thickness}} \right) +1 \end{aligned}$$

**Fig. 1:**
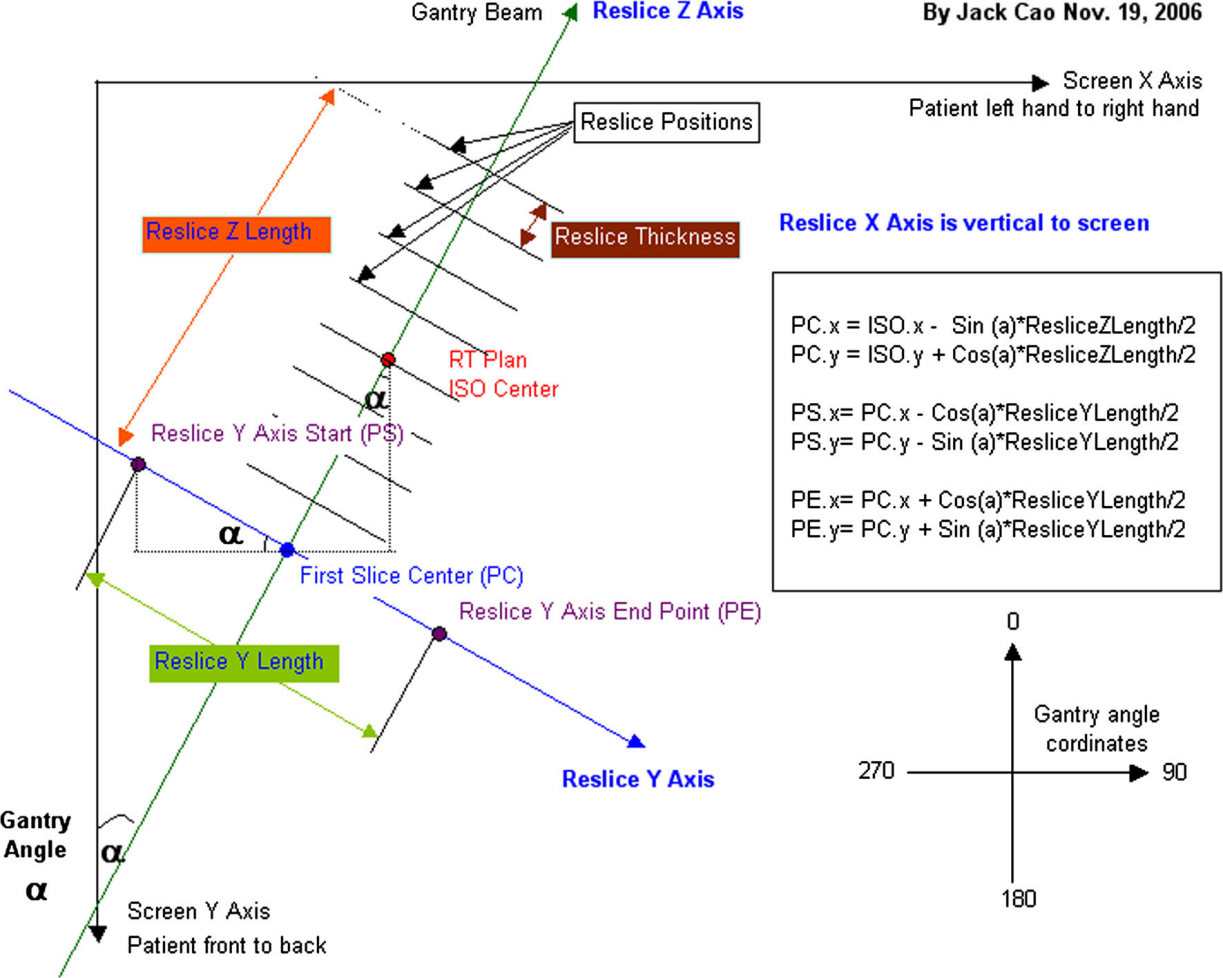
*Vision*’s reslice image calculations [8]

**Fig. 2:**
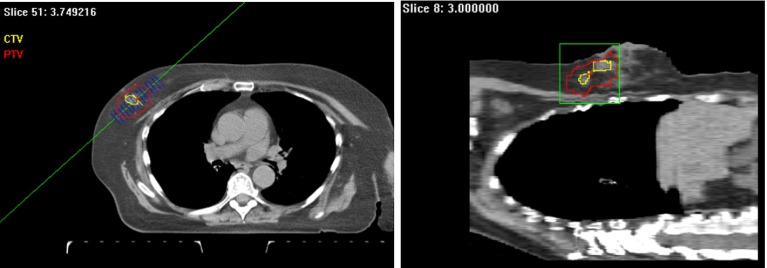
*Vision*’s reslice views: original data (*left*), and reconstructed from the left image (*right*)

The original data set is recalculated from the view that the gantry angle is specified by oncologist and is suitable for seed placement procedure, see Figs. 1 and 2.

### Volume interpolation

The interpolation component is designed to reconstruct image data and treatment volumes from the original slice thickness and spacing (can be any size) into 1 mm in spacing. The reconstructed target volume is built from the new set of contours—original plus interpolated contours. We assign the spacing equal to the number of interpolations. Therefore, the new resolution is always equal to 1 mm. With 5 mm spacing between two adjacent slices, four interpolated images and contours are created to achieve 1 mm of unit spacing.

**Fig. 3:**
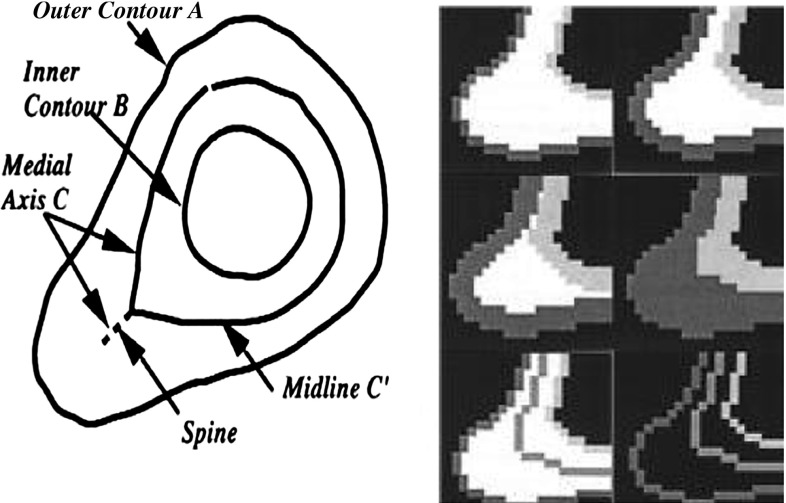
Morphological interpolation process [17]

When creating interpolation contours, two types of interpolation process are encountered, overlapped and intersected types. The intersect type is that one contour is inside the other; the overlapped type is that one contour partially inside the other. The interpolation contour is the dividing line that is equally dividing the region between two contours when only one interpolation is required. Figure 3 describes the mathematical operation for deriving the dividing line.

### Probability function for volume interpolation

When 2D contours are used to define treatment volumes, a partially subjective process is underlined [18]. The accuracy of volume definition depends on numerous image parameters such as the contrast to noise ratio and the signal-to-noise ratio. The subjective process and imaging parameters might cause inaccurate target volume creation. The volume definition using 2D contours is especially difficult at the edges of the target because of the partial volume effect that is related mostly to the image slice thickness [18]. A low inter- and intra-observer reproducibility was reported in different clinical studies, mainly because of the subjective and objective factors [18]. Consequently, a probability function was designed in *Vision* to study the partial volume effect of the volume definition caused by the 2D contouring method. The function is presented below using 26 for the total number of voxels in the neighborhood:

$$\begin{aligned} P=\frac{\mathrm{number}\, \mathrm{of}\, \mathrm{voxels}\,\mathrm{in}\, \mathrm{the}\, \mathrm{target}\, \mathrm{volume}}{26} \end{aligned}$$

**Fig. 4:**
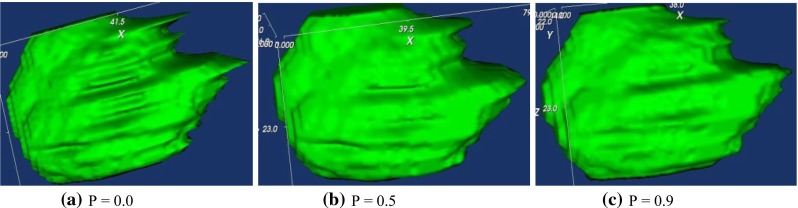
Volume refinement uses our 3D probability function; the sharp edges are removed with a degree that matches probability setting

The function is able to measure how many voxels belong to PTV in the neighborhood of current voxel. When the entire neighborhood of the current voxel belongs to the target volume, the probability value (output of the probability function) is 1; otherwise, it is 0. If part of the neighborhood voxels is inside the target volume and part is outside of the target volume, the probability value is between 0 and 1. A threshold value can be set for the probability function. The treatment volume is made up of all the voxels whose neighborhood probability value is greater than the threshold. When the threshold value is larger, the estimated volume is smaller (more neighborhood voxels are excluded from the target volume), see Fig. 4. The volumes generated using this probability function is more accurate than other methods when the threshold is set to 0.5.

### Radiation source configuration in vision

Task Group 43 (TG-43) from the American Association of Physicists in Medicine (AAPM) is currently used at the Toronto-Sunnybrook Regional Cancer Center [6]. Other kinds of radiation seeds (also called radial sources) can be used as radiation sources [6]. The radial sources are simulated by following AAPM TG-43 guidelines for brachytherapy to calculate dose distribution on different radiation sources. A source editor interface allows the user to input the radiation source and dose distribution charts. Currently, [$^{103}$Pd (2335) Best] is used for breast cancer brachytherapy [18].

### Virtual template implementation

**Fig. 5:**
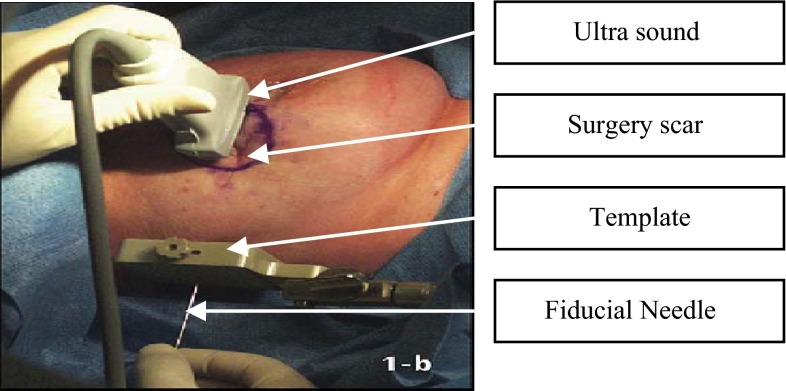
Seed implantation

Two templates guiding the insertion of needles loaded with seeds are simulated in *Vision*. A fiducial needle used to hook the surgical cavity, which is then attached to a template that guides the insertion of needles, is also simulated. Figure 5 shows the simulated template. Following image shows a real-time implantation operation using a needle one of the templates.

### Seed placement planning and dose distribution analysis interface

**Fig. 6:**
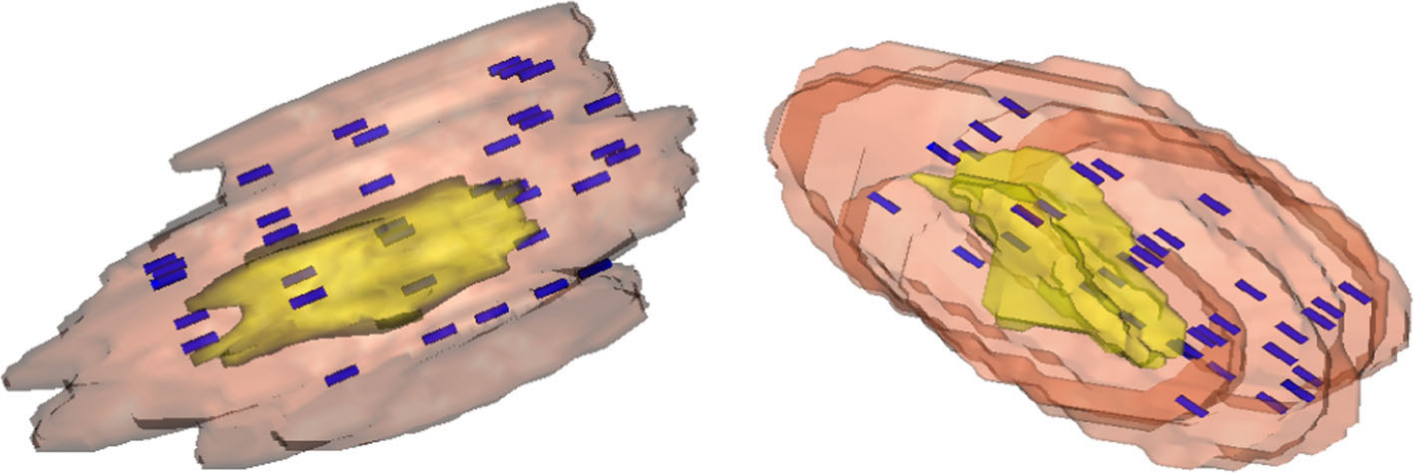
Virtual seed display on both patient’s PTV and CTV with random placed seeds

*Vision* provides an accurate, fast, and easy-to-use interface for seed placement on resliced CT data. Source placement spacing calculation in both 2D and 3D and dose volume histogram (DVH) is available. After seeds are placed on the resliced CT data set, a 3D view with the seeds can be generated for intuitive seed spacing analysis. This is a unique feature of *Vision* that does not exist among commercial brachytherapy treatment planning software systems. This virtual 3D environment displays the seeds in the original data set that provides an intuitive viewing environment. Figure 6 shows an example of the 3D volume rendering.

### Source editor: an interface for seed placement and dose calculation

**Fig. 7:**
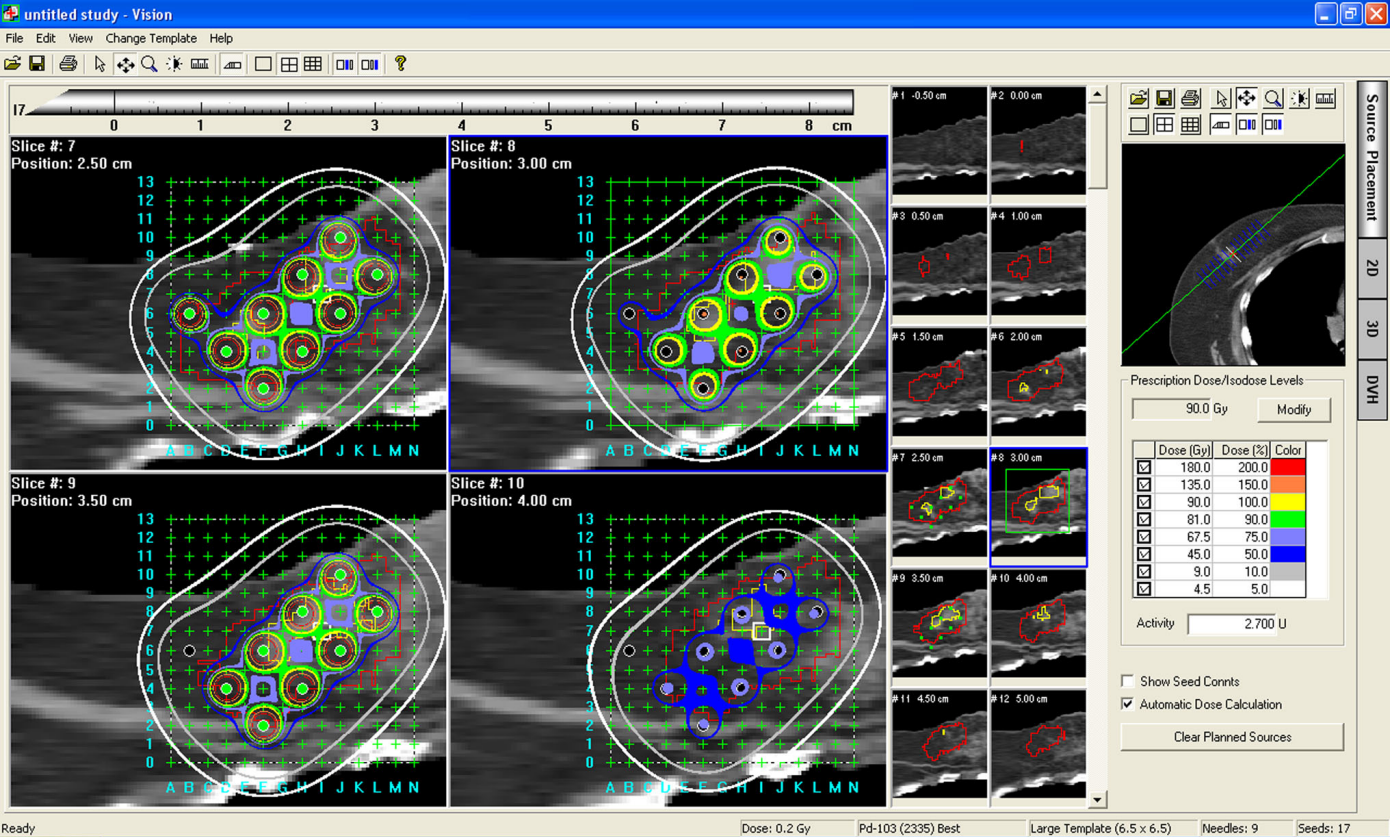
*Vision*’s user interface. Seed placement in 2D and 3D and dose volume histogram (DVH)

*Vision*’s source editor provides an interface that allows sources to be input and used. Currently, $^{103}$Pd (2335) Best is used for breast cancer brachytherapy. Figure 7 shows an example of source placement editing in 2D and 3D and its dose volume histogram (DVH). The dose at a particular point is calculated by summing the dose of all the seeds that affect the point. $^{103}$Pd (2335) Best seed is a cylindrically symmetric source, and dose distribution is a two-dimensional and can be described in terms of a polar coordinate system with its origin at the source center where $r$ is the distance to the point of interest and $q$ is the angle with respect to the long axis of the source.

The dose rate, $P(r,\theta )$, at point (r, $\theta$) can be written as follows:

$$\begin{aligned} P(r,\theta )=S_{k}^{\left[ \frac{G(r,\theta )}{G(ro,\theta o)}\right] g(r,\theta )},\hbox {where} \end{aligned}$$

$r$ is the distance to the point of interest, $\theta$ is the angle with respect to the long axis of the source, $S_{k}$ is air kerma strength of the source; $\left[ {\frac{G\left( {r,\theta } \right) }{G\left( {r0,\theta 0} \right) }} \right] g\left( r \right) F(r,\theta )$ is dose rate constant, $G(r,\theta )$ is geometry factor, $g(r)$ is radial dose function, $F(r, \theta )$ is anisotropy function.

### Reslice back with virtual seeds

One method of verifying the plan is by projecting the virtual implants back to their original data set. This innovative feature of our *Vision* system allows oncologists to view the implant position and spacing in 3D intuitively. In addition, *Vision* enables seed placement results to be viewed and validated.

## Results

We have tested the system using more than 30 data sets of breast cancer patients selected from the Toronto-Sunnybrook Regional Cancer Center after breast cancer treatment. Three phantom data sets were also tested.

**Fig. 8:**
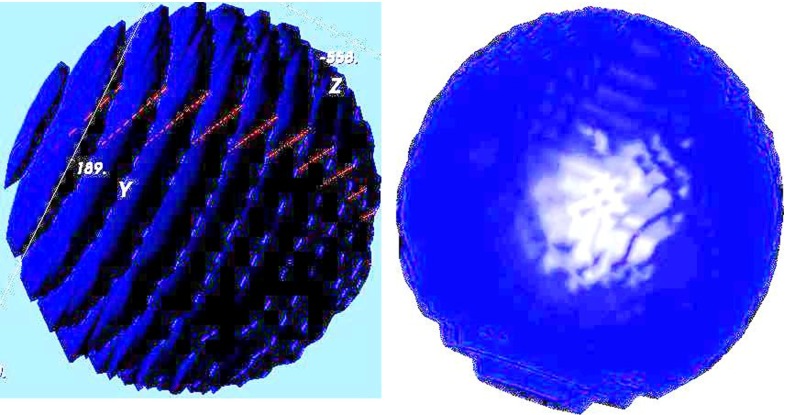
Plain target treatment volume 3D view, without interpolation (*left*), and with interpolation (*right*)

The dose distribution calculation is performed in real time while seeds are added. After seed implantation, 3D viewing of target volume containing all the placed seeds is displayed. The reslice calculation is also verified by projecting the image back to its original scanned direction. Our 3D reconstruction algorithm performs volume interpolation because of the non-uniform resolution of the scanned data sets. Normally, the in-plane ($X$–$Y$) resolution of a data set is greater than the out-of-plane ($Z$) resolution. This difference can range from a factor of 2–10. Our volume interpolation algorithm in *Vision* is able to adjust the number of interpolations between two slices automatically and then interpolate accordingly (see Fig. 8). The result spacing and thickness are always a unit of 1 mm between slices. Mathematical morphology operation for medial axis finding [17] and level set method [19, 20] are employed in the interpolation process.

**Fig. 9:**
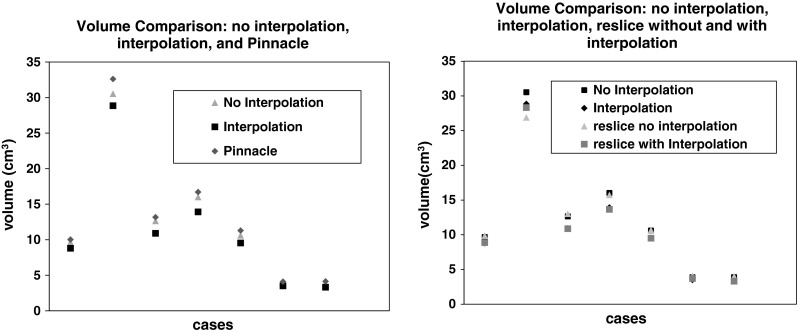
Volume output from existing software, with and without the interpolation method, and pinnacles’ output (*left graph*). Volumes comparison: no interpolation, interpolation, reslice no interpolation, and reslice with interpolation (*right graph*)

**Fig. 10:**
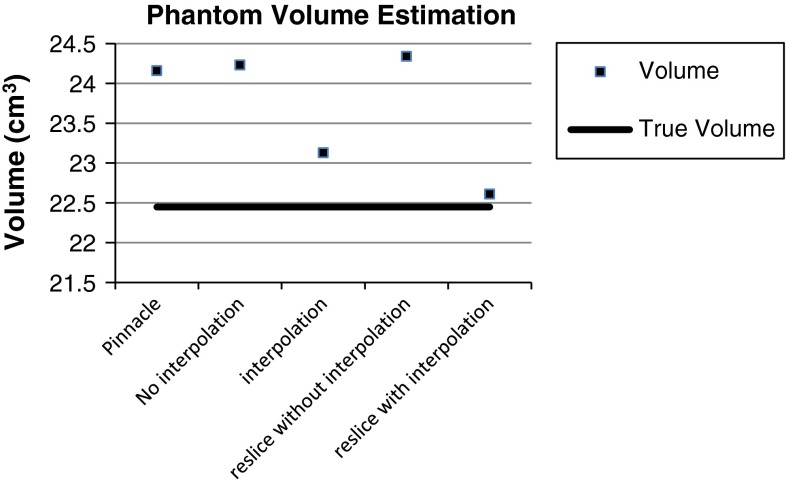
Phantom volume estimation: a comparison of several methods: pinnacle, no interpolation, interpolation (on original slices), reslice without interpolation, and reslice with interpolation

Our volume estimation method produces 99.73 % accuracy on sphere phantom data sets. When the probability threshold of volume estimation method is set to 0.5, the volume estimation gives the highest accurate volume estimation result. The result was compared with the Pinnacle system (Philips Medical imaging system). A statistically significant accuracy improvement on volume estimation was achieved (99.38 % from 92.38 % at the $p=0.05$ level). The true volume for this sphere phantom is as follows: 22.45 cm$^{3} \quad ({\mathrm{diameter}=3.5\,\mathrm{cm},\mathrm{volume}=\frac{4\pi 3^{3}}{3}}={22.45\,\mathrm{cm}^{3}})$. When using the interpolation method, more consistent volume estimation output is achieved. Our interpolation method provides the most accurate volume estimate as depicted in Fig. 9. Table 1 shows the testing results for seven data sets. The volume output from the existing commercial software overestimated the true volume (see Table 2; Figs. 9, 10).

**Table 1:** Volume estimations using patient CT data sets using different methods (in cm$$^{3})$$

Case no.	Pinnacle	No interpolation	Interpolation	Reslice without interpolation	Reslice with interpolation
1	10.01	9.66	8.78	9.86	8.85
2	32.59	32.52	28.83	26.90	28.30
3	13.15	12.64	10.89	13.02	10.86
4	16.69	16.01	13.90	15.74	13.65
5	11.28	10.60	9.53	10.67	9.48
6	4.09	3.86	3.49	4.08	3.70
7	4.12	3.89	3.31	4.01	3.30

**Table 2:** Volumes (in cm$$^{3}$$) and reconstructed volumes after reslice by our method with interpolation and without interpolation on phantom object with known volume (22.45 cm$$^{3})$$

Pinnacle	No interpolation	Interpolation	Reslice without interpolation	Reslice with interpolation
24.16	24.23	23.13	24.34	22.61

## Conclusions

This paper described *Vision*, a 3D computer-assisted treatment planning system we developed for breast cancer brachytherapy treatment. The system was developed using mathematical theories for accurate volume estimation and dose analysis, as well as advanced 3D visualization technologies. The patient treatment volume reconstructed by a method developed in this project significantly improved the accuracy from existing methods and guarantees the high-volume accuracy requirement of radioactive seed implantation procedure for this treatment. The *Vision* system is a virtual 3D environment that allows radiation oncologists to perform volume measurement, seed placement, and dose distribution planning and analysis based on a set of 2D contours on patient CT images. *Vision* is able to embed placed seeds on original patient CT images and displayed in an institutive 3D environment that can be easily manipulated by the oncologist.

Using our 3D neighborhood probability function to estimate the treatment volume, we were able to rectify the redundant voxels located outside of the target region and minimize the discrepancies in volume definition by 2D manually contouring between adjacent slices. In this research project, advanced mathematical theories were researched and applied, such as mathematical morphology theory, partial differential equations, probability statistics, and mathematical modeling. Our system achieves 99.73 % accuracy in volume estimation measured against the true volume and is statistically significantly more accurate than current existing commercial software at the $p=0.05$ level.

Future work will focus on automatic source seed placement that could be adjusted by an oncologist if necessary. The purpose of this work was to optimize the seed placements such that radial dose distributions will be uniformly distributed over the entire PTV to avoid hot/cold spots. This, in turn, will assist an oncologist by minimizing the number of seeds required and potentially increasing the effectiveness of the treatment.

## References

[1] Xiang H, Zhuang T (2002) CT/MRI-based system for 3-D brachytherapy treatment planning. In: 24th annual conference and the annual fall meeting of the biomedical engineering society EMBS/BMES conference, pp 1196–1197

[2] Hariri S (2010) Asl AK introducing a complementary treatment planning software for GZP6 brachytherapy system. In: 3rd international conference on biomedical engineering and informatics, pp 16–18

[3] Fisher B, Anderson S, Bryant J, Margolese RG, Deutsch M, Fisher ER, Jeong JH, Wolmark N (2002). Twenty-year follow-up of a randomized trial comparing total mastectomy, lumpectomy, and lumpectomy plus irradiation for the treatment of invasive breast cancer. N Engl J Med.

[4] Kuske RR, Young SS (2013). Breast brachytherapy versus whole-breast irradiation: reported differences may be statistically significant but clinically trivial. Int J Radiat Oncol Biol Phys.

[5] National_Cancer_Institute (2014) Surveillance, Epidemiology, and End Results Program (SEER)—Cancer of the Breast. http://seer.cancer.gov/statfacts/html/breast.html. Accessed April 10, 2014

[6] Pignol J-P, Keller B, Rakovitch E, Sankreacha R, Easton H, Que W (2006). First report of a permanent breast $$^{103}$$Pd seed implant as adjuvant radiation treatment for early-stage breast cancer. Int J Radiat Oncol Biol Phys.

[7] Zhang T, Patel R (2011) Optimization-based dosimetry planning for brachytherapy. In: 33rd annual international conference of the IEEE EMBS, Boston, Massachusetts, USA, 2011. IEEE, pp 5569–5572 10.1109/IEMBS.2011.6091347 22255601

[8] Jiang RY (2007) Software tool for breast cancer brachytherapy planning using VTK. Paper presented at the 6th IEEE international conference on cognitive informatics, Lake Tahoe, USA

[9] Curran D, van Dongen JP, Aaronson NK, Kiebert G, Fentiman IS, Mignolet F, Bartelink H (1998). Quality of life of early-stage breast cancer patients treated with radical mastectomy or breast-conserving procedures: results of EORTC Trial 10801. The European Organization for Research and Treatment of Cancer (EORTC), Breast Cancer Co-operative Group (BCCG). Eur J Cancer.

[10] van Dongen JA, Voogd AC, Fentiman IS, Legrand C, Sylvester RJ, Tong D, van der Schueren E, Helle PA, van Zijl K, Bartelink H (2000). Long-term results of a randomized trial comparing breast-conserving therapy with mastectomy: European Organization for Research and Treatment of Cancer 10801 trial. J Natl Cancer Inst.

[11] Poulin E, Fekete C-AC, Létourneau M, Fenster A, Pouliot J, Beaulieu L (2004) Virtual planning of multicatheter brachytherapy implants for accelerated partial breast irradiation. In: 26th annual international conference of the engineering in medicine and biology society conference. IEEE, pp 1–5 10.1109/IEMBS.2004.1403882 17270941

[12] Aneja S, Yu JB (2014). Comparative effectiveness research in radiation oncology: stereotactic radiosurgery, hypofractionation, and brachytherapy. Semin Radiat Oncol.

[13] Smith GL, Jiang J, Buchholz TA, Xu Y, Hoffman KE, Giordano SH, Hunt KK, Smith BD (2014). Benefit of adjuvant brachytherapy versus external beam radiation for early breast cancer: impact of patient stratification on breast preservation. Int J Radiat Oncol Biol Phys.

[14] Stewart AJ, Khan AJ, Devlin PM (2010) Partial breast irradiation: a review of techniques and indications. Br J Radiol 83(989):369–378 10.1259/bjr/11505970 PMC3473572 20223911

[15] Vargo JA, Verma V, Kim H, Kalash R, Heron DE, Johnson R, Beriwal S (2014). Extended (5-year) outcomes of accelerated partial breast irradiation using mammosite balloon brachytherapy: patterns of failure, patient selection, and dosimetric correlates for late toxicity. Int J Radiat Oncol Biol Phys.

[16] Foppiano F, Guatelli S, Moscicki J (2003) Pia MG From DICOM to GRID: a dosimetric system for brachytherapy born from HEP. In: Nuclear science symposium conference record. pp 1746– 1750

[17] Barrett W, Mortenson E, Taylor D (1994) An image space algorithm for morphological contour interpolation. Paper presented at the graphics interface conference

[18] Caudrelier JM, Vial S, Gibon D (2003) MRI definition of target volumes using fuzzy logic method for 3D conformal radiation therapy. Int Radiat Oncol Biol Phys 55(1):225–233 10.1016/s0360-3016(02)03829-4 12504057

[19] Sapiro G (2001). Geometric partial differential equations and image analysis.

[20] Sethian JA (1999) Level set methods and fast marching methods: evolving interfaces in computational geometry, fluid mechanics, computer vision, and materials science. Cambridge Press, Cambridge.

